# Dead or gone? Bayesian inference on mortality for the dispersing sex

**DOI:** 10.1002/ece3.2247

**Published:** 2016-06-21

**Authors:** Julia A. Barthold, Craig Packer, Andrew J. Loveridge, David W. Macdonald, Fernando Colchero

**Affiliations:** ^1^ Department of Zoology University of Oxford South Parks Road Oxford OX1 3PS UK; ^2^ Department of Public Health Max‐Planck Odense Center on the Biodemography of Aging University of Southern Denmark J.B. Winsløws Vej 9B, 5000 Odense C Denmark; ^3^ Max Planck Institute for Demographic Research Konrad‐Zuse‐Str. 1 18057 Rostock Germany; ^4^ Department of Ecology, Evolution and Behavior University of Minnesota 1987 Upper Buford Circle Saint Paul Minnesota 55108; ^5^ Department of Mathematics and Computer Science Max‐Planck Odense Center on the Biodemography of Aging University of Southern Denmark Campusvej 55 5230 Odense M Denmark; ^6^ Present address: Department of Public Health Max‐Planck Odense Center on the Biodemography of Aging University of Southern Denmark J.B. Winsløws Vej 9B 5000 Odense C Denmark

**Keywords:** African lion, age‐specific mortality, dispersal, sex differences in mortality, Siler model, true survival

## Abstract

Estimates of age‐specific mortality are regularly used in ecology, evolution, and conservation research. However, estimating mortality of the dispersing sex, in species where one sex undergoes natal dispersal, is difficult. This is because it is often unclear whether members of the dispersing sex that disappear from monitored areas have died or dispersed. Here, we develop an extension of a multievent model that imputes dispersal state (i.e., died or dispersed) for uncertain records of the dispersing sex as a latent state and estimates age‐specific mortality and dispersal parameters in a Bayesian hierarchical framework. To check the performance of our model, we first conduct a simulation study. We then apply our model to a long‐term data set of African lions. Using these data, we further study how well our model estimates mortality of the dispersing sex by incrementally reducing the level of uncertainty in the records of male lions. We achieve this by taking advantage of an expert's indication on the likely fate of each missing male (i.e., likely died or dispersed). We find that our model produces accurate mortality estimates for simulated data of varying sample sizes and proportions of uncertain male records. From the empirical study, we learned that our model provides similar mortality estimates for different levels of uncertainty in records. However, a sensitivity of the mortality estimates to varying uncertainty is, as can be expected, detectable. We conclude that our model provides a solution to the challenge of estimating mortality of the dispersing sex in species with data deficiency due to natal dispersal. Given the utility of sex‐specific mortality estimates in biological and conservation research, and the virtual ubiquity of sex‐biased dispersal, our model will be useful to a wide variety of applications.

## Introduction

Mortality estimates of both sexes for wild animal populations are fundamental for testing hypotheses derived from ecological and evolutionary theory, and for predicting population size and structure for population management purposes. However, estimating mortality of at least one of the sexes is commonly hindered by incomplete data on dispersing individuals. For example, in many large mammal species, males leave their natal place or social group around the age of maturity, while females are philopatric. If individuals of the dispersing sex, in this case males, leave the areas monitored by field studies that collect resighting data on marked individuals, these dispersing individuals impede the quality of gathered data in the following way.

Dispersing individuals are usually unavailable for collecting age‐at‐death data because following dispersing individuals using telemetry or GPS technology is costly and labor‐intensive. Furthermore, for many species deaths are rarely observed in the field. Instead, deaths are inferred from permanent disappearances of individuals from the study area. However, missing members of the dispersing sex, which were old enough for dispersal, may have died or dispersed. This uncertain fate of disappeared members of the dispersing sex makes the estimation of the mortality difficult using existing methods. The estimation of mortality for the philopatric sex is in comparison relatively straightforward because missing members of the philopatric sex are likely dead, even if their bodies are not found, as these individuals do not disperse.

Models to infer mortality using capture–mark–recapture/resighting (CMRR) data derived from the Cormack–Jolly–Seber framework (CJS; after Cormack 1964; Jolly 1965; Seber 1965) can accommodate both uncensored and right‐censored records (i.e., individuals known to be alive after the last observation). These approaches exploit the fact that each type of record contributes different information (White and Burnham 1999). Extensions to the initial models have been developed that accommodate species‐specific life histories and data issues arising from the movement of the individuals in relation to the spatial and temporal distribution of the marking and resighting effort. Accordingly, these models, known as multistate models (Arnason 1973; Schwarz et al. 1993), incorporate incomplete and heterogeneous resighting probabilities, multiple states, and multiple locations (e.g., Lebreton and Pradel 2002; Mackenzie et al. 2009; Cubaynes et al. 2010). Pradel (2005) extended the multistate framework to account for unobservable states, particularly in the context of movement between sites. This extension, known as multievent models, incorporates the estimation of uncertain states into the modeling of survival while accounting for dispersal rates and site fidelity (Avril et al. 2012; Lagrange et al. 2014). Alternatively, Ergon and Gardner (2014) extended the CJS model into a robust‐design spatial capture–recapture (RD‐SCR) model to jointly model survival and dispersal where the activity centers are treated as a latent state. Similarly, Schaub and Royle (2014) have recently developed a spatially explicit Cormack–Jolly–Seber approach that jointly models mortality and dispersal using movement data for species in which dispersal can be described as a random walk.

These approaches provide a fundamental framework to estimate survival under state uncertainty, particularly in the context of dispersal. Further complications arise when information on sex or ages is missing. In order to address issues with missing records in CMRR data, Bayesian approaches have been developed that estimate survival probabilities and transition probabilities between states and locations while augmenting data (Dupuis 1995, 2002; King and Brooks 2002). Some of these approaches estimate latent (unknown) states jointly with all other model parameters in a hierarchical framework using Markov chain Monte Carlo (MCMC) algorithms (Clark et al. 2005; Colchero and Clark 2012; Colchero et al. 2012). As latent states can be both finite sets of discrete states (e.g., locations or stages) or continuous variables (e.g., date of birth or death), this framework is suitable for developing a survival model that treats dispersal as a latent state, and can therefore accommodate uncertain records due to natal dispersal. This is particularly important for data sets where individuals of one or both sexes disperse but information on their movements is missing. In such cases, there is no information of the fate of potential dispersing individuals at the last time they are detected. At this time, their *dispersal state* (i.e., either dispersed or died) is unknown, and thus, the estimation of survival can be biased if this latent state is not explicitly modeled.

Here, we present a Bayesian hierarchical model that builds upon the multievent framework (Pradel 2005) and that estimates age‐specific mortality and dispersal for species where one sex is philopatric and one sex undergoes natal dispersal. The model fits a parametric age‐specific mortality model as a function of age and sex jointly with the estimation of the distribution of ages at dispersal, treating potential dispersal as a latent state. Using simulated data, we first validated the model. We then applied the model to estimate age‐specific mortality of both sexes for Serengeti lions (*Panthera leo*) in Tanzania. As this particular data set contains the expert opinion from the head of the study (C. Packer, unpublished data) on whether a missing male is likely to have dispersed or died, we used this information to gain further insights into the workings of our method. In particular, using the expert's opinion, we varied whether missing males entered the model as potential or known dispersers, and compared the mortality estimates among the different models in order to evaluate the influence of the imputation of dispersal as a latent state on our mortality estimates. For simplicity, we will refer to the philopatric sex as being female, and to the dispersing sex as being male. However, the model is flexible as to which sex is the dispersing sex, while it can be extended for the case where both sexes disperse.

## Methods

We focus on species in which individuals disperse out of the study area only once at around the age of maturity (“natal dispersal”) and where information on individual dispersal events is unavailable. In addition, our model is developed for data sets where movements within the study area are missing. To isolate the effect of uncertainty in male records on mortality estimates from other effects, we focus on data that meet the following assumptions. We assume that individuals are resighted with certainty if they are alive and in the study area. For estimating the age‐specific probabilities of dispersal for the dispersing sex, we further assume that mortality in‐ and outside the study area is equal and that individuals born outside the study area disperse into the study area with equal probabilities as individuals born in the study area disperse out of it. We also assume that ages of individuals whose birth was not observed (left‐truncated records) can be estimated with sufficient certainty by a trained observer to allow us to not include time of birth as a latent state in the model and to model ages at death as a continuous variable, although this can be included following Colchero and Clark (2012); Colchero et al. (2012). However, as the data available to us for the empirical application contained individuals that died before sexing was possible, we did construct the model to accommodate this type of record, treating the sex of unsexed individuals as another latent state. Finally, we further make one assumption that we know is not met for data from wild animal populations, and that is that mortality only depends on age and sex and not on any other covariates. However, this assumption allows us to develop a model to estimate baseline mortality for pooled data, which can later on be easily extended to incorporate other covariates.

### Life history data

#### Data structure

**Figure 1:**
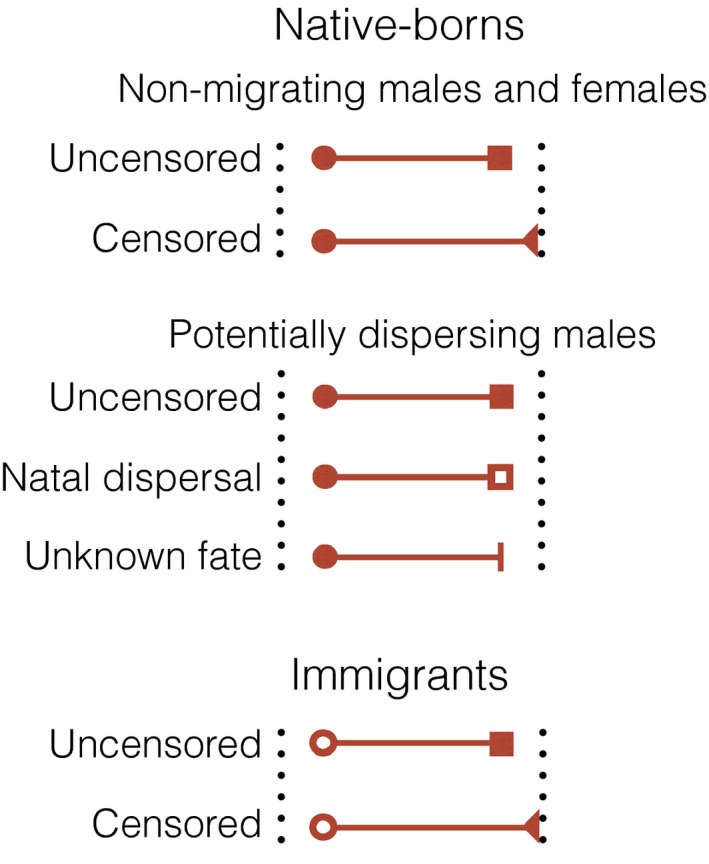
Example of types of records in the lion data set. Circles represent times of entry ($t_{i}^{F}$), where the entry type for filled circles corresponds to known times of birth and open circles are entries after birth (i.e., immigration or birth before the study started). Squares are departure times ($t_{i}^{L}$) where filled squares are known times of death and open squares are dispersal. Filled triangles indicate individuals known to be alive at the end of the study and vertical bars indicate that the type of departure from the study population is uncertain (i.e., either death or dispersal).

The life history data used to estimate age‐ and sex‐specific mortality included records for native‐borns and immigrants. Native‐borns were born in the study population, defined as all individually recognizable and constantly monitored individuals. Immigrants entered the study population some time after their birth due to migration (Fig. 1). Similarly, individuals that were located in the study area at the time the study began had a first detection age $x_{i}^{F}\;>\;0$. The recorded types of departure from the population included death, censoring due to being alive at the end of the study, or uncertain fate (death or censoring through dispersal). Uncertain fates through dispersal were only caused by dispersals from the study population to an external population, and not by dispersals within the study population. Here, we refer to this out‐migration from the study population when we use the term “dispersal.”

#### Serengeti population

The study population occupied a 2000 km${}^{2}$ region of Serengeti National Park, Tanzania, that lies at the heart of the Serengeti–Mara ecosystem. The study site is characterized by seasonal rainfall and a southeast to northwest gradient in vegetation from short to tall grassland to open woodlands (Packer 2005; Mosser et al. 2009). We analyzed demographic data collected between 1966 and 2013. Observations were opportunistic between 1966 and 1984, and most animals were sighted 1–3 times per month. Study prides have been monitored with radio telemetry since 1984, allowing each animal to be observed 2–6 times per month. All individuals are identified from natural markings (Packer et al. 1991), and birth dates of cubs born in the study area are deduced from lactation stains on the mothers. A large number of nomadic males enter the area, and a small proportion become resident in one or more of the resident prides. Our analyses exclude all nomadic males that never became residents in the study area (*N* = 548, ∼25% of all observations on males). These left‐truncated and right‐censored records contain little survival information. As a consequence, a model that included these records did not converge. Individuals with unknown dates of birth were assigned an estimated age by a trained observer, using age indicators (e.g., relative body size, nose coloration, and eruption and wear of teeth) (Smuts et al. 1978; Whitman et al. 2004). The data set contained a large number of individuals of unknown sex. As the vast majority of these unsexed individuals died within the first weeks after birth, we excluded all individuals with last detection ages younger than 0.25 years of age. The final data set contained observations on 1341 females, 1263 native‐born males, 316 immigrants, and 269 unsexed native‐born individuals. The proportion of females among all native‐born individuals (excluding immigrants), assuming a sex ratio of 1 to 1 among individuals that died before their sex could be determined, was 0.51.

### Mortality analysis

#### Model variables and functions

We developed a model that estimates both age‐specific mortality and dispersal where the dispersing state is unknown. Thus, at the time of last detection the dispersing state, $d_{i}$, for an individual *i* that belongs to the dispersing sex is treated as a latent state that needs to be estimated. Accordingly, the model requires defining random variables and probability functions for the age at death, *X*, and for the age at natal dispersal, *Y*, as well as for the binary latent state, *D*, with support given by $d_{i}\;=\;1$ if an individual is imputed to have dispersed and $d_{i}\;=\;0$ otherwise. Furthermore, we have extended the model to account for unknown sex, *S* (see Table 1 for a summary of all random variables, parameters, and indicators).

**Table 1:** Description of random variables, observed variables, and indicators

Modeled random variables	
*X*	Random variable for age at death, where *x* is any age element
*Y*	Random variable for age at natal dispersal with elements *y*
*D*	Binary random variable for disperser or nondisperser
*S*	Binary random variable for sex
Observed variables and indicators	
$t^{F}$	Vector of times of first detection
$t^{L}$	Vector of times of last detection
**b**	Vector of times of birth
$x^{F}$	Vector of ages at first detection ($x_{i}^{F}\;=\;t_{i}^{F}-b_{i}$)
$x^{L}$	Vector of ages at last detection ($x_{i}^{L}\;=\;t_{i}^{L}-b_{i}$)
**m**	Indicator vector for immigrants ($m_{i}\;=\;1$ if immigrant)
Updated indicators	
**d**	Indicator vector for dispersers ($d_{i}\;=\;1$ if disperser and $d_{i}\;=\;0$ otherwise)
**s**	Indicator vector for sex ($s_{i}\;=\;1$ if female and $s_{i}\;=\;0$ otherwise)
Parameters	
***θ***	Vector of mortality parameters
***γ***	Vector of natal dispersal parameters
Functions	
Mortality	
*μ*(*x*¦***θ***)	Mortality (Siler model)
*S*(*x*¦***θ***)	Survival
*F*(*x*¦***θ***)	CDF for age at death (*F*(*x*) = 1−*S*(*x*))
*f*(*x*¦***θ***)	PDF for age at death
Dispersal	
*g*(*y*¦***γ***)	PDF for age at natal dispersal (gamma distribution)
*G*(*y*¦***γ***)	CDF for age at natal dispersal

The age‐specific mortality model requires defining the mortality function or hazard rate as

(1) $$\mu \left(x\theta \right)=\lim_{\Delta x\to 0}\frac{\mathrm{Pr}(x\leq X<x+\Delta x\;\;x\leq X,\theta )}{\Delta x},\;x\geq 0$$

where ***θ*** is a vector of mortality parameters to be estimated. The estimated mortality can be used to calculate the probability of survival from birth to age *x*, or survival function,

(2a) $$S\left(x\theta \right)=\text{Pr}\left(X\geq x\right)=\exp\left[-\int_{0}^{x}\mu (z\theta )dz\right],$$

the probability that death occurs before age *x*, or the cumulative density function (CDF),

(2b) F(xθ)=Pr(X<x)=1−S(xθ),

and the probability density function (PDF) for age at death

(2c) $$f\left(x\theta \right)=\frac{d}{dx}F\left(x\theta \right)=S\left(x\theta \right)\mu \left(x\theta \right).$$

To capture the bathtub‐shaped mortality rates typical of large mammals, we used the Siler model (Siler 1979) in the form

(3) $$\mu \left(x\theta \right)=e^{a_{0}-a_{1}x}+c+e^{b_{0}+b_{1}x},$$

where $\theta^{⊤}=\left[a_{0},a_{1},c,b_{0},b_{1}\right]$, with $a_{0},b_{0}\in R$ and $a_{1},c,b_{1}>0$. The Siler model is a competing risk model constituted by three additive mortality hazards. The parameters capture different aspects of the shape of the age trajectory with the exponential of $a_{0}$ being the initial level of mortality rates and $a_{1}$ governing the exponential decrease in mortality over infant and juvenile ages. The *c* parameter scales mortality rates up or down and is usually interpreted as reflecting age‐independent causes of mortality. This parameter is also dominant in capturing mortality in early adult ages when infant mortality has declined and senescence mortality not yet risen. The exponential of the $b_{0}$ parameter represents the initial mortality of the age‐dependent increase of mortality and $b_{1}$ determines the rate of this increase (Siler 1979).

To model the ages at dispersal, we defined the random variable *Y* for age at dispersal, where the age at natal dispersal was $Y∼G_{Y}\left(y\right)$ for ages *y* > 0, with $G_{Y}\left(y\right)$ being the Gamma distribution function with parameter vector $\gamma^{⊤}=\left[\gamma_{1},\gamma_{2}\right]$. This distribution yields the probability density function (PDF) of age at natal dispersal given by

(4) $$g_{Y}\left(y\gamma \right)=\left\{\begin{array}{c} \frac{\gamma_{1}^{\gamma_{2}}}{\Gamma (\gamma_{2})}{\left(y-\alpha \right)}^{\gamma_{2}-1}e^{-\gamma_{1}\;\left(y-\alpha \right)}\;\text{if}\;y\geq \alpha \\ 0\;\text{if}\;y\geq \alpha , \end{array}\right.$$

where *α* is the minimum age at natal dispersal and $\gamma_{1},\gamma_{2}\;>\;0$.

At the age of last detection, $x^{L}$, individuals belonging to the dispersing sex can have dispersed, with a probability conditioned on *X* and *Y* given by

(5a) $$\mathrm{Pr}\left\{D=1x^{L}\right\}=\mathrm{Pr}\left\{X>x^{L}\wedge Y=x^{L}\right\}.$$

It is the joint probability that these individuals have not died and have dispersed shortly after the last detection age. The probability that these individuals have died and have not dispersed, but would have dispersed at later ages, is accordingly

(5b) $$\mathrm{Pr}\left\{D=0x^{L}\right\}=\mathrm{Pr}\left\{X=x^{L}\wedge Y>x^{L}\right\}.$$

As we specify above, the dispersal state is treated as a latent state and is therefore imputed. Below we explain how the likelihoods are specified and how the latent states are imputed. A summary of all the functions, parameters, indicators, and variables is provided in Table 1. R code to simulate data and fit the model can be downloaded from the link provided in the Supporting Information.

#### Likelihood and posterior

To construct the mortality likelihood, we assigned a different probability to each type of record in Figure 1. The likelihood for the nondispersing individuals (i.e., members of the nondispersing sex or members of the dispersing sex that disappeared at ages younger than the minimum age at dispersal *α*) is given by

(6a) $$p\left(x^{F},x^{L}\;\;\theta \right)=\left\{\begin{array}{c} \mathrm{Pr}(X=x^{L}\;\;X>x^{F},\theta )\text{if uncensored}\\ \mathrm{Pr}(X>x^{L}\;\;X>x^{F},\theta )\text{if censored,} \end{array}\right.$$

where $x^{L}$ corresponds to the age at last detection and $x^{F}$ is the age at first detection (i.e., $x^{F}\;=\;0$ for individuals born in the study area and $x^{F}\;>\;0$ for immigrants or individuals that were located in the study area when the study began). As we mentioned above, we defined dispersal state for all members of the dispersing sex with last seen ages older than the minimum age at dispersal *α* as a random variable *D*. It took the value $d_{i}\;=\;1$ if an individual *i*, born at $b_{i}$ and last detected at $t_{i}^{L}$, dispersed in its last detection age, $x_{i}^{L}\;=\;t_{i}^{L}-b_{i}$, and 0 if otherwise. For some individuals, $d_{i}$ is known either because the individuals were known to be alive and in the study area at the end of the study (i.e., right‐censored observations), or because their disappearance was known to be a death or a dispersal. For all other individuals, $d_{i}$ was imputed as a latent state.

Based on equations (5), the joint mortality and dispersal likelihood for members of the dispersing sex with $x_{i}^{L}\;>\;\alpha$ is given by

(6b) $$\begin{array}{c} p(x^{F},x^{L}\;\;d_{i},\theta ,\gamma )\\ \left\{\begin{array}{c} \mathrm{Pr}\left(X=x^{L},Y>x^{L}\;\;X>x^{F},\theta ,\gamma \right)\text{if uncens.}\;\;d_{i}=0\\ \mathrm{Pr}\left(X>x^{L},Y>x^{L}\;\;X>x^{F},\theta ,\gamma \right)ifcens.d_{i}=0\\ \mathrm{Pr}\left(X>x^{L},Y=x^{L}\;\;X>x^{F},\theta ,\gamma \right)ifd_{i}=1\\ \mathrm{Pr}\left(x^{F}<X=x^{L},Y=x^{F}\;\;\theta ,\gamma \right)\text{if}\;m_{i}=1, \end{array}\right. \end{array}$$

where $m_{i}$ is an indicator for individuals that joined the study population as immigrants, and thus, these individuals contribute important information on ages at death and dispersal.

Furthermore, we also defined a binary variable *S* for the sex of the individual. With this, we could construct the full Bayesian model as

(7) $$\begin{array}{c} p\left(d_{u},s_{u},\theta ,\gamma \;\;d_{k},s_{k},x^{F},x^{L}\right)\propto \underset{\text{likelihood}}{\underbrace{p(d_{k},s_{k},x^{F},x^{L}\;\;d_{u},s_{u},\theta ,\gamma )}}\\ \times \underset{\text{priors for states}}{\underbrace{p(d)\;p(s)}}\\ \times \underset{\text{priors for parameters}}{\underbrace{p\left(\theta \theta_{p}\right)\;p\left(\gamma \gamma_{p}\right)}} \end{array}$$

where **d** was the vector of dispersal states and **s** was the indicator vector for sex ($s_{i}\;=\;1$ if female and $s_{i}\;=\;0$ if male), and $\theta_{p}$ and $\gamma_{p}$ are vectors of prior hyperparameters for the mortality and dispersal parameters. Each of these vectors had two subsets represented by the subscripts *u* for unknown and *k* for known.

#### MCMC and conditional posteriors

We used a Markov chain Monte Carlo (MCMC) algorithm to fit the model in equation (7). For all implementations, we ran four parallel MCMC sequences with different randomly drawn starting values and set the number of iterations to 15,000 steps with a burn‐in of 5000 initial steps and a thinning factor of 20. We used a hierarchical framework that only needed the conditionals for posterior simulation by Metropolis sampling (Metropolis et al. 1953; Gelfand and Smith 1990; Clark 2007). This means that for this particular case, the algorithm divided the posterior for the joint distribution of unknowns into four sections: (a) estimation of mortality parameters, (b) estimation of dispersal parameters, (c) estimation of unknown dispersal state, and (d) estimation of unknown sexes. Here, we present each section, specifying the conditional posterior and the acceptance probability for the Metropolis Sampler algorithm.

##### Section a: Posterior for mortality parameters

The conditional posterior to estimate the mortality parameters ***θ*** required only the ages at first and last detection $x_{i}^{F}$ and $x_{i}^{L}$ and the dispersal states $d_{i}$. The posterior for a given individual *i* was

(8) $$p\left(\theta \;\;x_{i}^{L},x_{i}^{F},d_{i}\right)\propto \left\{\begin{array}{c} \frac{f(x_{i}^{L}\;\;\theta )}{S(x_{i}^{F}\;\;\theta )}\;\;p\left(\theta \theta_{p}\right)\text{if}\;d_{i}=0\\ \frac{S(x_{i}^{L}\;\;\theta )}{S(x_{i}^{F}\;\;\theta )}\;\;p\left(\theta \theta_{p}\right)\;\text{if}\;d_{i}=1\;\text{or} \end{array}\right.$$

where $\theta_{p}$ was a vector of prior hyperparameters. If the individual was a native‐born, then $x_{i}^{F}\;=\;0$ and the denominator in both expressions was equal to 1. At every iteration and for a given parameter *θ* ∈ ***θ*** with conditional posterior *p*(*θ*¦⋯), the algorithm proposes a new parameter value for each element of $\theta^{\prime }$ and accepts it with acceptance probability

(9) $$p\left(\theta ,\theta^{\prime }\right)=\min\left\{1,\frac{\prod_{i=1}^{n}p\left(\theta^{\prime }\;\;x_{i}^{L},x_{i}^{F},d_{i}\right)}{\prod_{i=1}^{n}p\left(\theta \;\;x_{i}^{L},x_{i}^{F},d_{i}\right)}\right\}.$$

##### Section b: Posterior for dispersal parameters

The conditional posterior to estimate the parameters ***γ*** for the distribution of ages at natal dispersal for a given individual *i* was

(10) $$p\left(\gamma \;\;x_{i}^{F},x_{i}^{L},d_{i},\omega_{i},m_{i}\right)\propto \left\{\begin{array}{c} g\left(x_{i}^{L}-\alpha \;\;\gamma \right)\;\;p\left(\gamma \;\;\gamma_{p}\right)\;\text{if}\;\omega_{i}=1,\;m_{i}=0\;d_{i}=1\\ \left[1-G\left(x_{i}^{L}-\alpha \;\;\gamma \right)\right]\;p\left(\gamma \;\;\gamma_{p}\right)\;\text{if}\;\omega_{i}=1,\;m_{i}=0\;d_{i}=0\\ \frac{g(x_{i}^{F}-\alpha \;\;\gamma )}{S(x_{i}^{F}\;\;\theta )}\;\;p\left(\gamma \;\;\gamma_{p}\right)\text{if}\;m_{i}=1\\ \text{otherwise}, \end{array}\right.$$

where $\gamma_{p}$ was a vector of prior hyperparameters for ***γ***, $\omega_{i}$ was an indicator that assigns 1 if an individual was a potential disperser (i.e., if it belonged to the dispersing sex and disappeared at an age older than the minimum age at dispersal *α*), and $m_{i}$ was an indicator for immigrants. We set the minimum age at dispersal to *α* = 1.75 years for the simulated data and *α* = 1.5 for the Serengeti data. The age *α* corresponded to the earliest age at which immigrants could be detected and potential dispersers could be last seen. For a parameter *γ* ∈ ***γ*** with conditional posterior density *p*(*γ*⋯), the acceptance probability for a proposed parameter of $\gamma^{\prime }$ was

(11) $$p\left(\gamma ,\gamma^{\prime }\right)=\min\left\{1,\frac{\prod_{i=1}^{n}p\left(\gamma^{\prime }\;\;\cdots \right)}{\prod_{i=1}^{n}p\left(\gamma \;\;\cdots \right)}\right\}.$$

##### Section c: Posterior for dispersal states

Dispersal state was evaluated for individuals that were potential dispersers (i.e., $\omega_{i}\;=\;1$). The joint probabilities for dispersal state were

(12) $$p\left(d_{i}\;\;x_{i}^{L},\omega_{i},m_{i}\right)\propto \left\{\begin{array}{c} f\left(x_{i}^{L}\right)\left(1-G\left(x_{i}^{L}\right)\right)\;\;p\left(d_{i}\theta_{p},\gamma_{p}\right)\text{if}\;\omega_{i}=1,m_{i}=0,d_{i}=0\\ S\left(x_{i}^{L}\right)g\left(x_{i}^{L}\right)\;\;p\left(d_{i}\theta_{p},\gamma_{p}\right)\text{if}\;\omega_{i}=1,m_{i}=0,d_{i}=1\\ -1080\;\text{otherwise}. \end{array}\right.$$

The first terms on the right‐hand side of equation (12) correspond to the likelihood function as defined in equations (6), while the second terms are the priors for dispersal state. For this section, the acceptance probability for the sampling given the last detection ages, the dispersal states, the potential disperser states, and the immigration states was

(13) $$p\left(d_{i},d_{i}^{\prime }\right)=\min\left\{1,\frac{\prod_{i=1}^{n}p\left(d_{i}^{\prime }\;\;x_{i}^{L},\omega_{i},m_{i}\right)}{\prod_{i=1}^{n}p\left(d_{i}\;\;x_{i}^{L},\omega_{i},m_{i}\right)}\right\}.$$

##### Section d: Posterior for unknown sexes

Some individuals disappeared before the minimum age at dispersal without their sex being determined. The conditional posterior for the latent state of sex was

(14) $$p\left(s_{i}\;\;x_{i}^{L},\theta \right)\propto p\left(x_{i}^{L},\theta \;\;s_{i}\right)\;\;p\left(s_{i}\right),$$

where the second term on the right‐hand side is a prior for sex based on the sex ratio at birth, or if the analysis was conditioned on survival to age *x*, based on the sex ratio at age *x*.

The indicator for potential dispersers $\omega_{i}$ (see Section c) was updated in each iteration. Individuals of undetermined sex and last detection ages older than the minimum age at dispersal were assigned 1 if imputed to be male and 0 if imputed to be female. The acceptance probability given the last detection ages and the mortality parameters was

(15) $$p\left(s_{i},s_{i}^{\prime }\right)=\min\left\{1,\frac{\prod_{i=1}^{n}p\left(s_{i}^{\prime }\;\;x_{i}^{L},\theta \right)}{\prod_{i=1}^{n}p\left(s_{i}\;\;x_{i}^{L},\theta \right)}\right\}.$$

#### Mortality and dispersal priors

We set the Siler parameters for the prior for both sexes to $a_{0p}\;=\;-3$ (*σ* = 0.5), $a_{1p}\;=\;0.2$ (*σ* = 0.25), $c_{p}\;=\;0$ (*σ* = 0.25), $b_{0p}\;=\;-4$ (*σ* = 0.5), and $b_{1p}\;=\;0.01$ (*σ* = 0.25). For dispersal, the Gamma parameters (shape and scale) for the prior were set to $\gamma_{p}\;=\;\left\{8,2\right\}$ with $\sigma \left(\gamma_{p}\right)\;=\;\left\{2,1\right\}$. Priors were normally distributed and truncated at 0, apart from the level parameters of the Siler model ($a_{0}$ and $b_{0}$), which were not truncated. Both the mortality and dispersal priors were fairly uninformative. The priors for the probability of being female was 0.5 for the simulated data and 0.51 for to the Serengeti data (see also subsection “Serengeti population”).

#### Model application and posterior analysis

We fitted the model to the Serengeti data with sex as a covariate, which was imputed for individuals with unknown sex. We included the covariate by making the mortality parameters contained in ***θ*** functions of the covariate, namely

(16) $$\theta_{i}=\theta_{i_{1}}s_{i}+\theta_{i_{2}}\left(1-s_{i}\right),$$

where $s_{i}\;=\;1$ if female and 0 otherwise.

In order to gain deeper insights into the performance of our model, we further exploited a unique source of information that is contained in this data set. A Serengeti lion expert used the circumstances accompanying the disappearances of males to deduce whether the individuals may have dispersed (C. Packer, unpublished data). For example, as young males often leave their natal prides with brothers, a simultaneous disappearance of brothers hints that this is likely to be a dispersal event. We fitted the model with three different settings. First, all males with uncertain fates and last detection ages older than minimum age at dispersal were assigned the state of “potential dispersers” and entered in the model as described in “Section c” above (Model A). Second, all males that were indicated by the expert to potentially have dispersed were entered as “known dispersers” (see equation (6b)b) (Model B). And third, all males that were indicated by the expert to potentially have dispersed were entered as “potential dispersers” while other uncertain male records were treated as having died at the last detection age (Model C).

To avoid problems arising from the large number of unsexed individuals that died within the first weeks after birth, we fitted the model from the start age of 0.25 years. We predicted mortality rates for each sex using the parameter estimates of every step of the MCMC after burn‐in and thinning and used these predictions to calculate mean and credible intervals of mortality rates. To compare the three models to each other, we computed the life expectancy at the model start age and the Kullback–Leibler (KL) divergences of the mortality parameter posterior densities (Kullback and Leibler 1951; McCulloch 1989; Burnham and Anderson 2001) (see Methods S1 for details on the calculation and the interpretation of KL values).

#### Simulated data

To validate the performance of our model, we used known mortality parameters to simulate data of the described structure and checked whether our model accurately retrieved these parameters. To simulate the data, we first randomly assigned a sex for an initial number of individuals by drawing from a binomial distribution, assuming an equal probability of being born male or female. We then randomly drew ages at death ($x_{i}$) for each individual *i* by inverse sampling from a Siler CDF (see equations (2b)b and (3)) with parameters $\theta_{f}\;=\;\left\{-1.4,0.65,0.07,-3.8,0.2\right\}$ for females and $\theta_{m}\;=\;\left\{-1.2,0.7,0.16,-3.5,0.23\right\}$ for males. The subscripts *f* and *m* denote females and males, respectively. We then randomly drew ages at dispersal for all males by inverse sampling from a gamma CDF with parameters ***γ*** = {10,3} and adding the minimum age of dispersal *α* = 1.75. We assigned every individual a last detection age $x_{i}^{L}$ depending on its sex and dispersal status. For females and for those males whose ages at death were simulated to be younger than their ages at dispersal (i.e., they died before they could disperse), the last detection ages were the ages at death. For the other males, who were simulated to have died after dispersal, the last detection ages were set to be the ages at dispersal. Finally, to add immigrants to the data, we simulated the same number of males being born in the external population. For these males, as before, we randomly drew ages at deaths and ages at dispersal, and if they were simulated to have dispersed before death, we added them to the data as immigrants with their ages at death recorded as last detection ages and their ages at dispersal recorded as first detection ages $x_{i}^{F}$.

We simulated data sets of two different initial numbers of native‐borns (small sample size *N* = 500 and large sample size *N* = 2000). Within each sample size, we also produced further data sets where the sexes of all individuals were known, and data sets where we randomly assigned, with a probability of 0.3, the state of “unknown sex” to all individuals that died at <1 year of age. Finally, we simulated data that varied in the proportion of observed or “known” deaths among individuals that were no longer resighted. We used three settings: 1, 5, and 10% known deaths. In total, we thus simulated 12 data sets. All simulations and analyses were conducted using the statistical computing language R (R Core Team 2012).

## Results

### Simulation study

**Figure 2:**
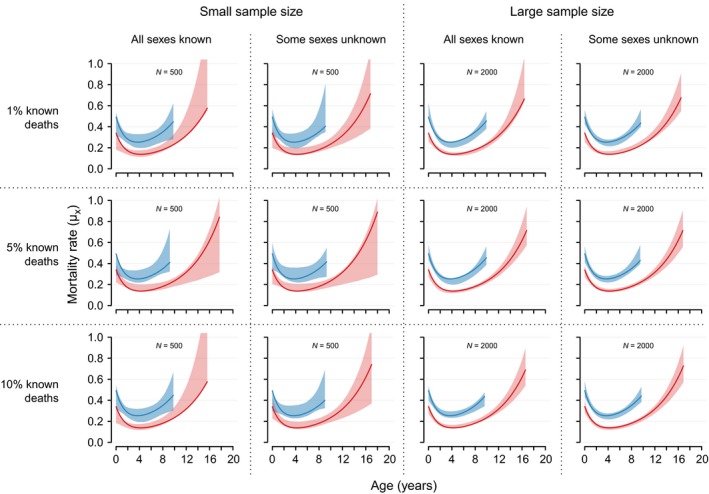
Predicted mortality rates for males (blue polygons) and females (pink polygons) compared to the mortality rates used to simulate the data (solid lines). Polygons represent 95% credible intervals of age‐specific mortality rates. Mortality rates are plotted until the ages when 95% of a synthetic same‐sex cohort would be dead. Results are given for 12 simulations varying the size of the native‐born population (*N* = 500 or *N* = 2000), the proportion of known deaths among last detection ages (1%, 5%, or 10%), and whether the sex of 30% of individuals dying younger than 1 year of age remained undetermined or not.

We used a simulation study to validate our model. For all 12 simulations, the mortality rates used to simulate the data lay within the 95% credible intervals of the estimated mortality for both sexes (Fig. 2). Of all the introduced variations in data quality (sample size, unsexed individuals, proportion “known” deaths), the only one with a marked effect on the performance of the model was varying the sample size. As could be expected, smaller sample sizes resulted in wider credible intervals particularly for males and for older ages of females. Due to the wider confidence bands for smaller sample sizes, the respective estimated mortality rates could appear to be less variable over the life span than the mortality rates used to simulate the data. This manifested as a less pronounced U‐shape of the estimated mortality rates when compared to the “real” mortality rates (e.g., second panel in second row of Fig. 2). The proportion of unsexed individuals dying at <1 year of age, and the proportion of known deaths among disappearances did not discernibly affect the retrieval of the mortality parameters.

### Application

**Figure 3:**
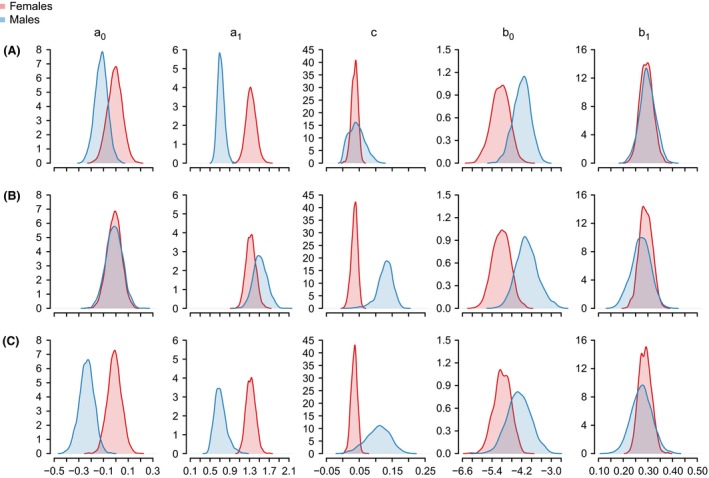
Posterior distributions of Siler parameter estimates ($a_{0}$, $b_{0}$, *c*, $a_{1}$, $b_{1}$) for female (pink) and male (blue) African lions of the Serengeti population. The analysis was conditioned on survival of the first 3 months of life.

**Figure 4:**
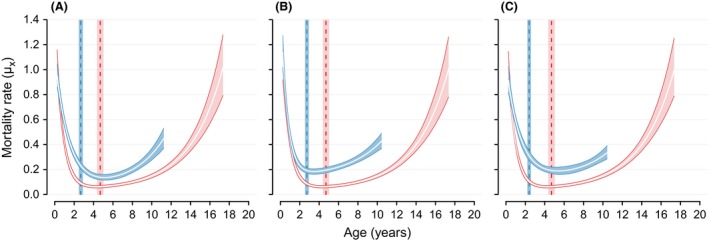
Age‐specific mortality estimates for male (blue lines and polygons) and female African lions (pink lines and polygons) of the Serengeti population. Polygons represent 95% credible intervals of age‐specific mortality rates with white lines indicating the mean. Mortality rates are plotted until the ages when 95% of a synthetic same‐sex cohort would be dead. The vertical dashed lines indicate mean life expectancy at 0.25 years of age with the 95% confident bands indicated by the rectangles. (A) Model A: all males with uncertain fate and old enough for dispersal treated as potential dispersers. (B) Model B: all males indicated by an expert as potential dispersers treated as known dispersers. (C) Model C: all males indicated by an expert as potential dispersers treated as potential dispersers.

The empirical models for Serengeti lions converged for all estimated parameters (Fig. 3; see also Figs. S1–S3 for traces). To supplement the visual inspection of the chains, we further confirmed convergence for the *c* parameters using the potential scale reduction (Gelman et al. 2013). We obtained values very close to 1 (between 0.999 and 1.002) for five of the six estimated *c* parameters (Model A, B, C and both sexes). Only one *c* parameter for females had a value of 1.05, which is still within the limits of having reached convergence. Overall mortality of both sexes was U‐shaped with high initial cub mortality, low mortality of prime‐aged adults, and an age‐dependent increase in mortality at older ages (Fig. 4). Mortality of males was higher than mortality of females across all ages (Fig. 4), except for very young ages, up until 1 year, during which confidence bands of male and female mortality overlapped. However, this may be due to the large proportion of unsexed individuals at these ages (see data description) and the imputation of sex as a latent state for these individuals, which introduced uncertainty. Due to the higher male mortality rates across most ages, female life expectancy (4.7 years at model start age) exceeded that of males by approximately 2 years.

Now we turn to the comparison between the models with varying settings for potential dispersers. Model A (Fig. 4A) treated the data as if no further information was available on dispersal status of males with uncertain fates (i.e., the default setting of the model). Model B took advantage of expert knowledge on lion behavior and treated all males that a lion expert believed were dispersers, as known dispersers (Fig. 4B). Finally, Model C treated all expert‐indicated potential dispersers as potential dispersers and thus considered all other uncertain male records to represent deaths (Fig. 4C). The number of potential dispersers whose dispersal state was imputed as a latent state was therefore smaller in Model C when compared to Model A.

**Figure 5:**
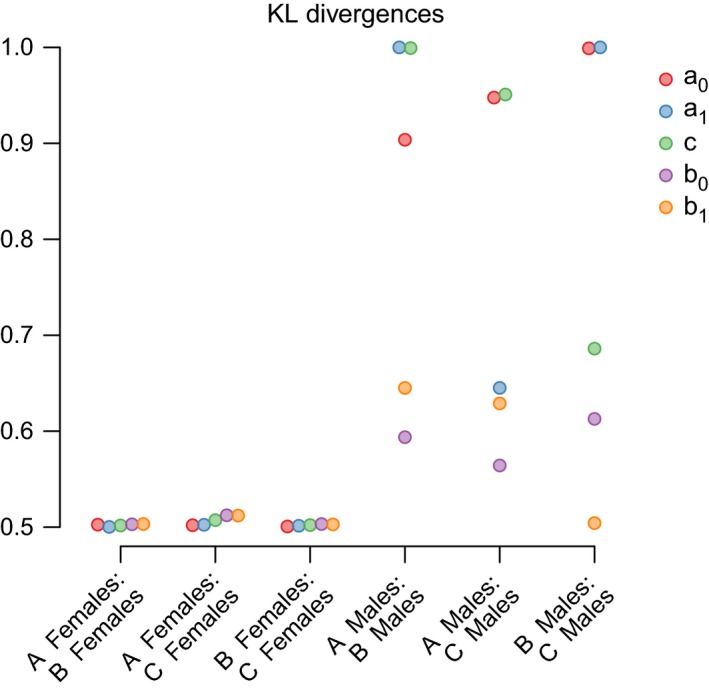
Kullback–Leibler (KL) divergences comparing same‐sex Siler parameter posteriors among the three models (A–C) with varying settings for males with uncertain fate. Note that the KL divergence estimates are jittered in *x*‐axis direction to improve visibility. The analysis was conditioned on survival of the first 3 months of life.

We compare these models by examining the estimated mortality rates (Fig. 4), the posterior density distributions (Fig. 3), and the KL divergences (Fig. 5). As females were treated the same way in all three models, the posterior distributions of parameters for females were congruent among the three models (Fig. 3). Consequently, the KL divergences were close to, or equal to, 0.5 (Fig. 5), and female mortality rates were almost identical across all three models (Fig. 4).

For males, the three models gave slightly varying results. The different settings regarding potential dispersers mostly affected the estimation of the Siler parameters that describe initial mortality ($a_{0}$), the age‐dependent decrease in mortality at young ages ($a_{1}$), and the age‐independent mortality (*c*) (Fig. 5). The initial mortality was higher in Model B, and lower in Model C, when compared to the default model A (Fig. 3, Table S1). The age‐dependent decrease in mortality was steeper in Model B compared to Model A but similar between Model A and C. The age‐independent mortality was higher in both Model B and C when compared to the default Model A.

The differences among the three models can be more fully understood by comparing the male mortality rates predicted from the three models (Fig. 4). Due to the steep decline in age‐dependent mortality at younger ages when all expert‐indicated dispersers were treated as dispersers (Model B), mortality rates during the juvenile ages up to approximately three years of age were lower in Model B when compared to both models that imputed dispersal state for potential dispersers (Model A and C). However, for the prime‐adult ages, Model B gave the highest mortality estimates, followed by Model C, and then Model A, which gave the lowest estimates. Mortality rates at older ages were highest in Model A and B. Despite these differences in the shape of the mortality rates curves, the life expectancies at 0.25 years of age were predicted to be identical by Model A and B (2.7 years), and only slightly different by Model C (2.4 years).

## Discussion

Life history data of wild animals are often incomplete because animals, even though alive and well, may temporarily or permanently be absent when researchers try to observe them at a given location. This has far reaching consequences for the estimation of biological properties from these data. Accordingly, various statistical approaches have been developed that account for temporal and spatial heterogeneity in recapture probabilities. For example, multistate CMRR methods have been applied to estimate survival rates while accounting for migration between locations within study sites (Arnason 1973; Schwarz et al. 1993; Lebreton and Pradel 2002; Pradel 2005; Mackenzie et al. 2009; Lagrange et al. 2014). And spatially explicit CMRR methods have been developed to estimate survival probabilities and population size (Borchers and Efford 2008; Efford and Mowat 2014; Ergon and Gardner 2014). Furthermore, a recently developed spatially explicit Cormack–Jolly–Seber approach jointly models dispersal and survival hierarchically for species in which dispersal movements can be assumed to follow a random walk (Schaub and Royle 2014).

However, these models require some information on movement within the study area to estimate mortality parameters and latent states. Our model is an alternative to these models for data sets where no information on movement within the study is available and thus dispersal state is entirely unknown. Instead, potentially dispersing individuals are resighted with certainty as long as they are alive and in the study area, and they are not resighted after they dispersed. To meet these challenges, our model does not model spatially heterogeneous detection probabilities and dispersal distances but rather imputes the dispersal state of the uncertain male records (i.e., died or dispersed) as a latent state variable in a Bayesian hierarchical framework (Clark et al. 2005; Colchero and Clark 2012; Colchero et al. 2012). We therefore show that for species with sex‐specific natal dispersal, mortality and dispersal can be jointly modeled without using movement data. Of course, movement data could potentially be used to inform the dispersal process. However, we decided to develop a model that does not rely on spatial data so that the model can easily be applied to data sets that differ in the structure of available spatial data.

To gauge the possibility of estimating sex‐ and age‐specific mortality in species with sex‐biased natal dispersal, we focused on data with incomplete records for sex and age at death. We assumed that this uncertainty could arise from one of two mechanisms. Firstly, native‐born males that disperse from the study area can cause uncertainty in male records of age at death, and secondly, individuals dying as juveniles before their sex could be determined resulted in uncertain sex records. Implicitly, the model therefore assumes that all birth dates are known and that all other types of records can be treated as complete records. Consequently, the model treated the last detection ages of potential dispersers that were imputed to be nondispersers and of immigrants as certain ages at death. The accuracy of the model therefore hinges on the assumption that potential dispersers disperse only once during their life. During our study, it became apparent that while this assumption holds for some lion populations (A. Loveridge, unpublished data), it does not hold for the Serengeti population.

**Figure 6:**
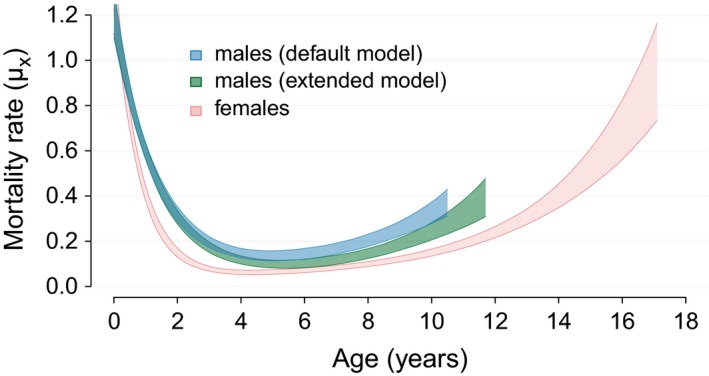
Age‐specific mortality estimates for male and female African lions of the Serengeti population. Polygons represent 95% credible intervals of age‐specific mortality rates. Mortality rates are plotted until the ages when 95% of a synthetic same‐sex cohort would be dead. The blue polygons represent male mortality rates obtained from the default model that accounts for natal dispersal. The green polygons represent male mortality rates obtained from an extended model, where secondary dispersal was accounted for additionally to natal dispersal by entering last detection ages of likely secondary dispersers as age of right‐censoring.

Relaxing the assumption and accounting for higher‐order dispersal necessitates a customized extension of the mortality model we present here. The effectiveness of fitting this more complex model depends on the availability of information on both known deaths and dispersal events among immigrants. In the case of the Serengeti population, we took advantage of the expert's indication on likely dispersal state of disappearing immigrants and extended the default model (Model A) to treat all immigrants that were indicated to be likely dispersers as censored at last seen ages. The difference between the male mortality estimates from the default model and the extended model provides an indication of the amount by which male mortality is overestimated if secondary dispersal is not accounted for (Fig. 6). To improve mortality estimates, in future extensions of the model secondary dispersal can be imputed as a further latent state, similarly to what we have showcased here for natal dispersal.

Another consequence of the treatment of immigrants’ last detection ages as ages at death is that the ratio of immigrants to dispersers is likely to influence the estimation of male mortality parameters. Problems may arise if the number of individuals that disperse out of the study area is much higher than the number of individuals that immigrate into it (see Fig. S4 for a simulation). This may be the case for field sites that are established in protected areas and act as a source population for surrounding habitats of lower quality. Mortality in these habitats, and mortality during the dispersal process itself, may also be higher than mortality within the study area. Our model cannot account for this heterogeneity because the data only contain information collected within the study area.

Finally, the comparison of the different models for the lion data allows us to draw some conclusions about the sensitivity of mortality estimates to varying levels of uncertainty in male records. If all expert‐indicated dispersers were in fact dispersers (Model B), then by comparing the mortality rates estimated by this model to the one with the default treatment of uncertain records (Model A), we learn that the default model may have the tendency to overestimate mortality during juvenile ages (lower $a_{1}$ in Model A than B). The default model may furthermore slightly underestimate mortality during prime‐adult ages. As the model that treats all expert‐indicated dispersers as potential dispersers and treats all other uncertain records as deaths (Model C) shares properties of both Models A and B (similar *c* to Model B, similar $a_{0}$ and $a_{1}$ to Model A), and may come closest to reality, it seems like a promising avenue for future development to directly include expert knowledge in the Bayesian framework via priors. However, this information is an idiosyncrasy of the data set that we used here. Making the model dependent on this information would therefore preclude the application of the model to estimate mortality for other populations and species.

In conclusion, we have discussed here how the model hinges on various assumptions. If these are met, then the model performs well at estimating mortality of the dispersing sex, as we have shown in the simulation study. The assumptions appear to restrict the utility of the model because many ecological data sets may not comply with them. However, we have explained how the different assumption can be relaxed by extending the basic, here‐presented model. The hierarchical framework and the modeling of the joint probabilities of ages at death and dispersal for potential dispersers provide flexibility that can be exploited to adapt the model to the specific data structure of each data set. Extensions can include other covariates, information on interval censoring, and imperfect detection probabilities. For example, an extension to account for secondary dispersal, dispersal of both sexes, and unknown times of birth is currently developed for a comparative study of six primate populations (F. Colchero, unpublished data). Overall, we conclude that our model provides a good solution to the challenge of estimating mortality of the dispersing sex in species with data deficiency for the dispersing sex due to natal dispersal.

## Conflict of Interest

None declared.

## Supporting information


**Figure S1–S3.** Traces of mortality and dispersal parameter estimation for Models A to C.
**Figure S4.** Predicted mortality functions for males (blue polygons) and females (pink polygons) compared to the mortality functions used to simulate the data (solid lines), if the probability of immigration into the study area of males born outside of it was lowered from 1 to 0.5.
**Table S1.** Estimated coefficients for Models A to C.
**Code S1.** R code to simulate data, to run the model on simulated data, and to plot the output can be downloaded from github.com/bartholdja/mortality-estimation-method.
**Methods S1.** Calculation and calibration of Kullback–Leibler divergence.
