# Supplementary material for: Dead or gone? Bayesian inference on mortality for the dispersing sex
Source: Ecol Evol. 2016 Jun 21;6(14):4910–23. doi: 10.1002/ece3.2247 (PMC4979716; doi:10.1002/ece3.2247)
Supplement: Supplementary file 1 — Figure S1–S3. Traces of mortality and dispersal parameter estimation for Models A to C. Figure S4. Predicted mortality functions for males (blue polygons) and females (pink polygons) compared to the mortality functions used to simulate the data (solid lines), if the probability of immigration into the study area of males born outside of it was lowered from 1 to 0.5. Table S1. Estimated coefficients for Models A to C. Code S1. R code to simulate data, to run the model on simulated data, and to plot the output can be downloaded from github.com/bartholdja/mortality-estimation-method. Methods S1. Calculation and calibration of Kullback–Leibler divergence. [file ECE3-6-4910-s001.pdf]

Supporting information

Supporting methods

Code S1:

R code to simulate data, to run the model on simulated data, and plot the output can be downloaded from github.com/bartholdja/mortality-estimation-method.

Methods S1: Calculation and calibration of Kullback-Leibler divergence

The Kullback-Leibler (KL) divergence calculates the difference or the amount of overlap between two distributions (Kullback and Leibler, 1951; McCulloch, 1989; Burnham and Anderson, 2001). To illustrate the calculation of KL, let's take a parameter θ, for which the resulting 'sub-parameters' for females and males would be θ_f and θ_m, respectively. Thus, for an individual i, we have $\theta = \theta_f I_i + \theta_m(1 - I_i)$, where I_i is an indicator function that assigns 1 if the individual is a female and 0 otherwise. For each of these parameters, our model produces a posterior distribution, say $P_f = p(\theta_f | \dots)$ and $P_m = p(\theta_m | \dots)$, respectively. The KL between these distributions is calculated as

$$K(P_f, P_m) = D_{f,m} = \int_{-\infty}^{\infty} P_f \, \log\left(\frac{P_f}{P_m}\right) \mathrm{d}\theta \, . \qquad \text{(S-1)}$$

The result can be interpreted as how far off we would be if we tried to predict θ_m from the posterior distribution of θ_f. If both distributions are identical, then $D_{f,m} = 0$, suggesting that there is no distinction between the parameters of both covariates and hence, that both covariates have the same effect. With increasing KL values, the discrepancy becomes higher. As can be inferred from Equation S-1, the relationship is asymmetric, namely $K(P_f, P_m) \neq K(P_m, P_f)$.

To make KL values easier to interpret, McCulloch (1989) proposed a simple calibration of the KL values that reduces the asymmetry. Let $k = K(P_f, P_m)$ and

q_k be a calibration function such that

$$\begin{aligned} k &= K(P_f, P_m) \\ &= K\left[B\left(\frac{1}{2}\right), B(q_k)\right], \end{aligned}$$

where $B(\frac{1}{2})$ is a Bernouilli distribution for an event with probability 0.5 (i.e., same probability of success and failure). This calibration is then calculated as

$$q_k = \frac{\left[1 + (1 - e^{-2k})^{\frac{1}{2}}\right]}{2}. \tag{S-2}$$

Thus, q_k ranges from 0.5 to 1, where a value of 0.5 means that the distributions are identical, and 1 that there is no overlap between them.

Supporting figures and tables

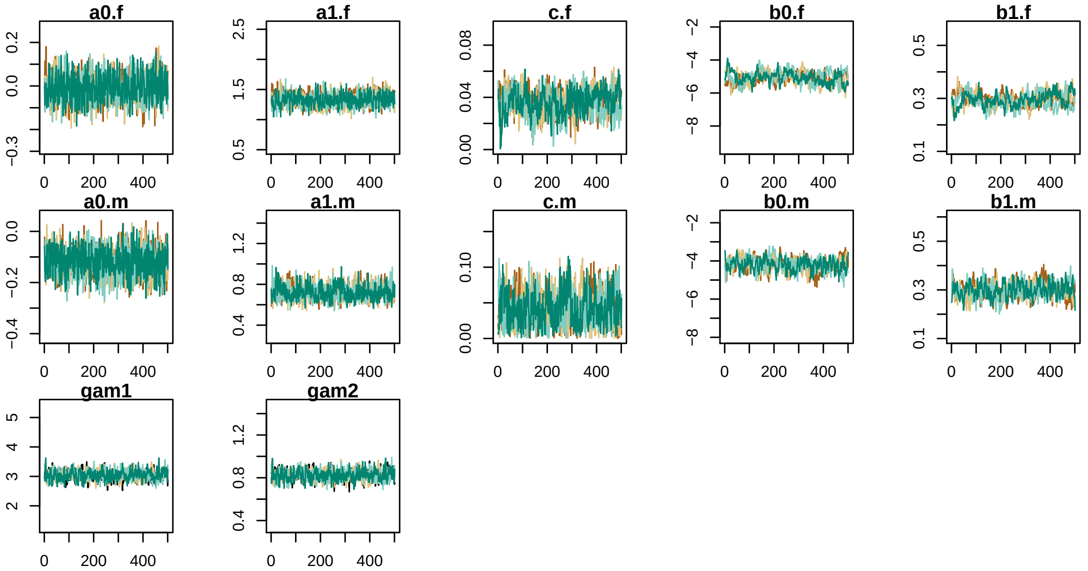

Figure S1: Trace plots for four parallel runs for the Serengeti lion mortality analysis. Estimated parameters are the Siler parameters (a_0, b_0, c, a_1, b_1; f denotes estimates for females and m for males) and Gamma parameters (shape and rate; gam1 and gam2). Model A : all males with uncertain fate and last seen ages older than minimum age at dispersal treated as potential dispersers.

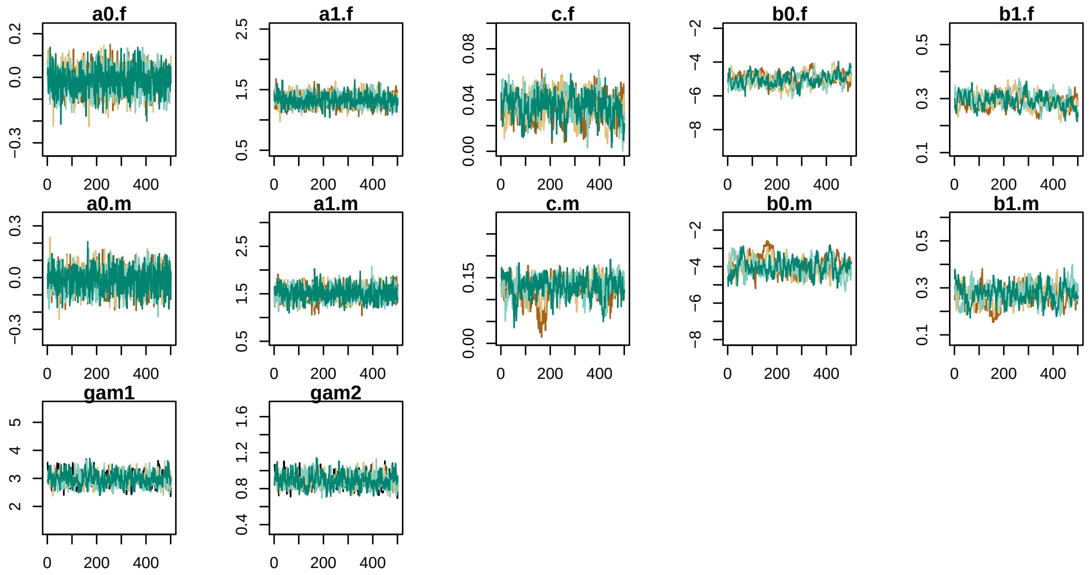

Figure S2: Trace plots for four parallel runs for the Serengeti lion mortality analysis. Estimated parameters are the Siler parameters (a_0, b_0, c, a_1, b_1; f denotes estimates for females and m for males) and Gamma parameters (shape and rate; gam1 and gam2). Model B : all males that an expert indicated as potential dispersers treated as known dispersers.

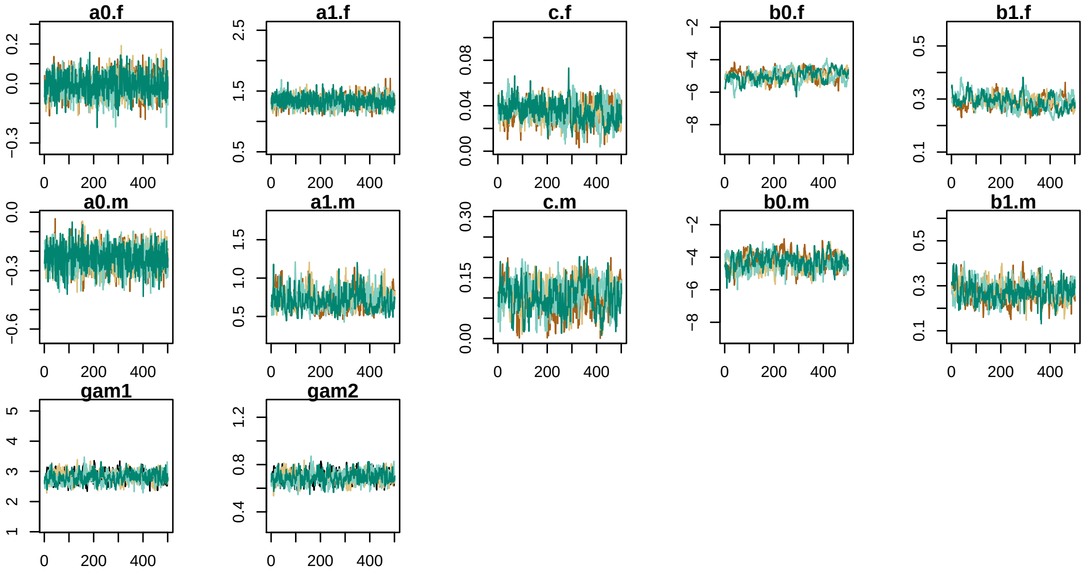

Figure S3: Trace plots for four parallel runs for the Serengeti lion mortality analysis. Estimated parameters are the Siler parameters (a_0, b_0, c, a_1, b_1; f denotes estimates for females and m for males) and Gamma parameters (shape and rate; gam1 and gam2). Model C: All males that an expert indicated as potential dispersers treated as potential dispersers.

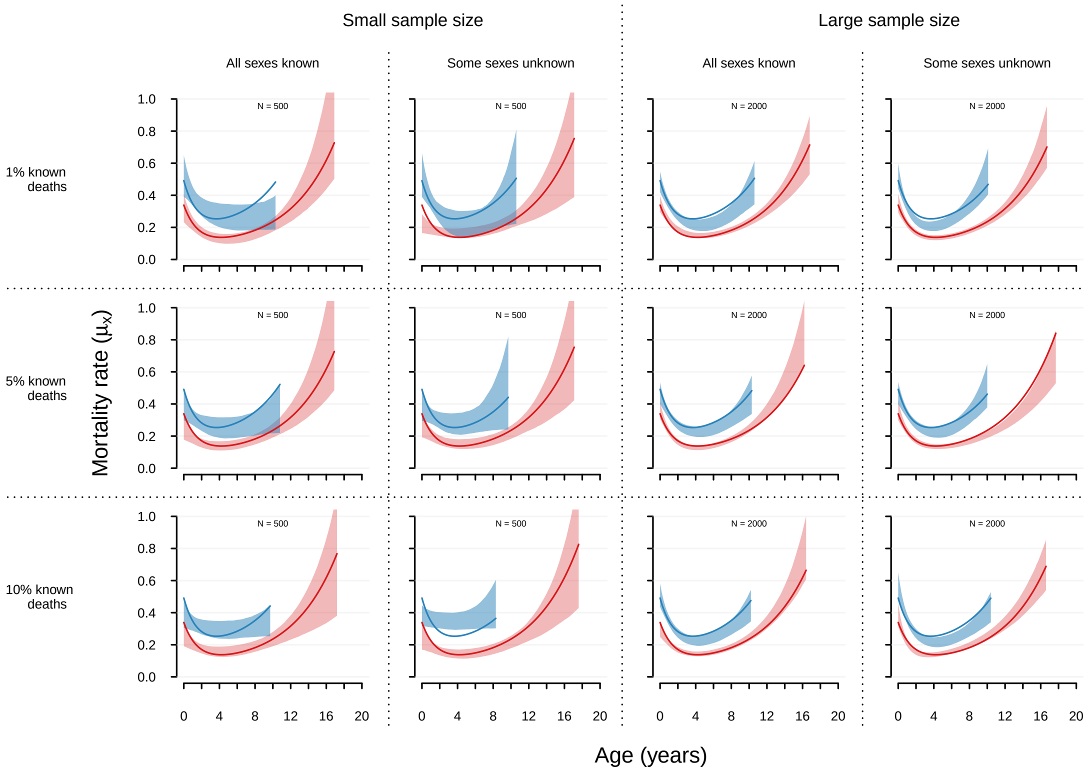

Figure S4: Predicted mortality functions for males (blue polygons) and females (pink polygons) compared to the mortality functions used to simulate the data (solid lines), if the probability of immigration into the study area of males born outside of it was lowered from 1 to 0.5. Polygons represent 95 % credible intervals of age-specific mortality functions. Mortality rates are plotted until the ages when 95 % of a synthetic same-sex cohort would be dead. Results are given for 12 simulations varying the size of the native-born population (N = 500 or N = 2000), the proportion of known deaths among last seen ages (1%, 5%, or 10%), and whether the sex of 30% of individuals dying younger than 1 year of age remained undetermined or not.

Table S1: Estimated Siler and gamma coefficients for Model A (all males with uncertain fate and old enough for dispersal treated as potential dispersers), Model B (all males indicated by an expert as potential dispersers treated as known dispersers, and Model C (all males indicated by an expert as potential dispersers treated as potential dispersers). Given are mean, SE, and credible intervals of the parameter posterior distributions.

		Coefficient	Mean	SE	2.5 %	97.5 %
Model A	Females	a_0	-0.01	0.06	-0.12	0.11
		a_1	1.33	0.10	1.14	1.54
		c	0.04	0.01	0.02	0.05
		b_0	-5.06	0.36	-5.79	-4.38
		b_1	0.29	0.03	0.24	0.35
	Males	a_0	-0.12	0.05	-0.22	-0.01
		a_1	0.72	0.07	0.60	0.87
		c	0.04	0.02	0.00	0.09
		b_0	-4.19	0.35	-4.89	-3.55
		b_1	0.30	0.03	0.24	0.36
		gam_1	3.04	0.17	2.72	3.37
		gam_2	0.82	0.05	0.73	0.92
Model B	Females	a_0	-0.01	0.06	-0.12	0.10
		a_1	1.33	0.10	1.14	1.53
		c	0.04	0.01	0.01	0.05
		b_0	-5.02	0.36	-5.70	-4.33
		b_1	0.29	0.03	0.24	0.34
	Males	a_0	-0.01	0.07	-0.14	0.12
		a_1	1.52	0.14	1.25	1.80
		c	0.13	0.02	0.07	0.17
		b_0	-3.98	0.44	-4.81	-3.03
		b_1	0.27	0.04	0.19	0.34
		gam_1	2.97	0.22	2.56	3.45
		gam_2	0.89	0.07	0.76	1.04
Model C	Females	a_0	-0.01	0.06	-0.12	0.10
		a_1	1.33	0.09	1.15	1.51
		c	0.03	0.01	0.02	0.05
		b_0	-4.99	0.34	-5.68	-4.35
		b_1	0.29	0.03	0.24	0.34
	Males	a_0	-0.24	0.06	-0.36	-0.12
		a_1	0.71	0.12	0.51	1.00
		c	0.11	0.04	0.03	0.17
		b_0	-4.31	0.47	-5.21	-3.34
		b_1	0.27	0.04	0.19	0.36
		gam_1	2.81	0.18	2.46	3.16
		gam_2	0.69	0.05	0.60	0.80

References

Burnham KP, Anderson DR. 2001. Kullback-Leibler information as a basis for strong inference in ecological studies. *Wildlife Research* **28**: 111–119.

Kullback S, Leibler RA. 1951. On information and sufficiency. *Annals of Mathematical Statistics* **22**: 142–143.

McCulloch RE. 1989. Local model influence. *Journal of the American Statistical Association* **84**: 473–478.
